# Silurian Climatic Zonation of Cryptospore, Trilete Spore and Plant Megafossils, with Emphasis on the Přídolí Epoch

**DOI:** 10.3390/life14020258

**Published:** 2024-02-16

**Authors:** Jiří Bek, Philippe Steemans, Jiří Frýda, Viktor Žárský

**Affiliations:** 1 Laboratory of Palaeobiology and Palaeoecology, Institute of Geology of the Academy of Sciences of the Czech Republic, 165 00 Prague, Czech Republic; 2 Eddy Lab/Palaeopalynology, Department of Geology, University of Liège, 4000 Liège, Belgium; p.steemans@ulg.ac.be; 3 Faculty of Environmental Sciences, Czech University of Life Sciences Prague, 165 21 Praha 6, Czech Republic; 4 Czech Geological Survey, 118 21 Prague 1, Czech Republic; 5 Faculty of Science, Charles University, 128 43 Prague 2, Czech Republic; viktor.zarsky@natur.cuni.cz

**Keywords:** early land plants, cryptospores, trilete spores, Přídolí, Silurian

## Abstract

This paper describes dispersed cryptospores and trilete spores from tropical, temperate and cool climate belts within Přídolí and compares them with the land plant megafossil record. The palynology of earlier intervals in the Silurian are also reviewed. A common feature of the cryptospore and trilete spore records is that their number is surprisingly lowest in the tropical climatic belt and much higher in the temperate and especially in the cool latitude, and the highest number of cryptospore taxa occurring only in one belt is found in the cool belt while the highest number of trilete spore taxa that occurred only in one belt is recorded in the temperate belt. In general, based on the dispersed spore record, we can estimate that the plant assemblages of the tropical belt were dominated by rhyniophytes; trimerophytes probably prevailed over rhyniophytes in the temperate belt, and rhyniophytes again dominated within the cool belt.

## 1. Introduction

This paper compares the occurrence of cryptospores, trilete spores and plant megafossil records during the Silurian in cool, temperate and tropical climate belts, with an emphasis on the Přídolí epoch, but also including earlier stages as far back as the Telychian. We characterize climate belts using palynology, and compare those to the plant megafossil record in the Pridoli (earlier stages having too sparse a megafossil record for comparison).

Plants are a critical element of life on the continents as we know it. Some authors have suggested that embryophytes (land plants) are monophyletic, that bryophytes and tracheophytes are sister clades based on critical molecular phylogenesis [1,2,3], and that land plants evolved from multicellular charophycean green algae [4,5,6]; surprisingly, recent theories emphasise the sister relationship of unicellular or filamentous Zygnematophyceae charophytes to land plants [7,8,9,10].

The oldest Silurian plant specimens are interpreted as allochthonous, mainly from coastal marine sediments [11] including volcanic islands [12,13,14,15]. The first indirect evidence for early land plants are cryptospores [16], which occur as monads, dyads and permanent tetrads and were produced by a heterogeneous group called Eophytidae [17], former cryptosporophytes [18] and cryptophytes. The first hypothetical palynomorphs that might be interpreted as cryptospores were described in the Middle Cambrian [19], consistent with phylostratigraphic predictions of the possible emergence of crown embryophytes [20], and the last specimens are from the Middle Devonian [2,21,22]. True trilete spores represent vascular land plants and their immediate ancestors [11,23,24], and the oldest trilete spores are reported from the Ordovician [25,26].

The oldest undoubtedly known sporophyte of a vascular land plant is *Cooksonia barrandei* Libertín et al. [12] from the middle Sheinwoodian strata of the Perunica microplate (Peri-Gondwana; now the Czech Republic; [13]). Other important events are peaks of cryptospore abundance within the Hirnantian–Rhuddanian interval [2]. The first global event for the diversification of early land plants and the change from cryptospore to hilate/trilete spore dominance is palynologically documented (trilete spores) after the Homerian glaciation [2,15]. The second significant and global event occurred after the Middle Ludfordian glaciation [27,28] during the Přídolí [2,21] and is defined by the combination of early land plant and trilete spore records. Also important is the appearance of monolete spores from the same locality as *C. barrandei* within the Sheinwoodian [15], i.e., 432 Ma.

A summary of Sheinwoodian, Homerian, Gorstian and Ludfordian cryptospore and trilete spore assemblages is given to illustrate trends of the pre-Přídolí palynological record. Sheinwoodian represents the minimum of both cryptospore and trilete spore diversity, and the first global event for cryptospore and mainly trilete spore producers was after the Homerian glaciation [2]; cryptospore and trilete spore diversity decreased in the Gorstian, and during the Ludfordian the diversity of both types of palynomorphs slightly increased, with the biggest change occurring in the Přídolí.

The Eophytidae (cryptospore producers) were probably not climatically sensitive and were cosmopolitan in nature, possibly preferring more arid areas away from water [29] suggest that Eophytidae preferred arid areas away from water. Early vascular land plants (trilete spore producers) were more widespread with a diversity of habits, without access to waterways and therefore to the sea [30], i.e., more climatically sensitive than Eophytidae. Plant invasions on land were characterised by limited competition and suitable environments would have been rapidly colonized [2]. Early land plants were homosporous and their isospores were easily transported over long distances, mainly by wind but also by water, although [30] suggest that long-distance dispersal was uncommon and rare.

Wind transport was probably the most important means of spores’ dispersal at that time. The majority of Silurian dispersed and in situ spores are in the 20–40 µm size range while the optimum wind dispersal is 25 µm [31]. Wind dispersal was easier and more suitable because the vegetation was at ground level, there were no tall plants to impede transport out of the boundary layer and into the wind current at higher latitudes, the average surface wind strength was stronger due to the absence of large vegetation, and the total atmospheric pressure was higher than it is today, resulting in a higher air density [32].

Significant changes in spore morphology (palynological events) are summarised in Table 1. Unfortunately, the Silurian plant megafossil record is much sparser than the spore record, rendering direct comparison problematic [2].

Palynological records from the Dapingian–Llandovery interval support the idea that vegetation was relatively uniform during these 30 Ma [2]. Refs. [33,34] reported dispersed spores from the Hirnantian glaciation, providing evidence for the cold tolerance of early land plants.

The record of trilete spores represents an easily recognisable group that is probably monophyletic, whereas cryptospores are difficult to work with because their distribution may be quite cosmopolitan and cryptospore records are often lacking in nearshore marine sediments [35].

Ordovician–Silurian dispersed spore assemblages have been reported by numerous palynologists, including [2,23,27,30,36,37,38,39,40,41,42]. Important sources of information are in situ spores, i.e., spores isolated directly from plant reproductive organs [43,44,45,46,47]. The plant record from the Ordovician–Silurian interval is limited and poor. The first plant megafossils were reported in the 1930s [48] and plant assemblages are still not numerous. Ref. [49] published an overview of Silurian plants in the form of 16 assemblages. Later, ref. [50] reported an analysis of Silurian plants based on 35 assemblages and recognised four phytogeographic units; North Laurussian (Bathurst Island), South Laurussian–SW Gondwana, Kazakhstanian and SW Gondwana.

Přídolí plant megafossils are much more diverse than those of stratigraphically older units [25]. For example, 10 plant genera with 13 species are known from Ludlow, but 16 plant genera with 24 species are reported from Přídolí [25]. This means that there is a significant change in plant assemblages between Ludlow and Přídolí, which is similar to an event described, for example, by [2,21,25].

The last significant summary of the early land plant record, mainly cryptospore and trilete spores, was published by [2] and this updated database is included here.

The biggest lower Paleozoic event for plants was in Přídolí [2,21,25] and the aim of this study is to determine which climate belt was the most suitable for the observed diversification of land plants.

For many years, the Silurian was thought to be the most important interval for the terestrialisation of early land plants. It now appears, also based on comparative phylostratigraphy, that the earliest land plants colonised the land as early as the Ordovician [51,52] or maybe even the Middle Cambrian [20]. However, the Silurian is the most important for more permanent expansion and more continuous plant cover.

The Silurian, which lasted from about 443.8 to 419.2 million years ago [53], represents a key interval in Earth’s history and biological evolution. During this time, vascular plants diversified and colonised the continents during the “Siluro-Devonian terrestrial radiation“, which can be considered the terrestrial equivalent of the “Cambrian explosion“ in marine life [54,55].

Přídolí is the youngest Series of the Silurian System, ranging from 423 to 419.2 Ma [53]. The name is derived from the Přídolí area of the Daleje Valley in the Czech Republic. The base is defined by the first appearance of the graptolite species *Monograptus parultimus* Jaeger. In addition, two species of chitinozoans, *Urnochitina urna* Eisenack and *Fungochitina kosovensis* Paris and Kříž, first occur at or just above the base of the Series.

**Figure 1:**
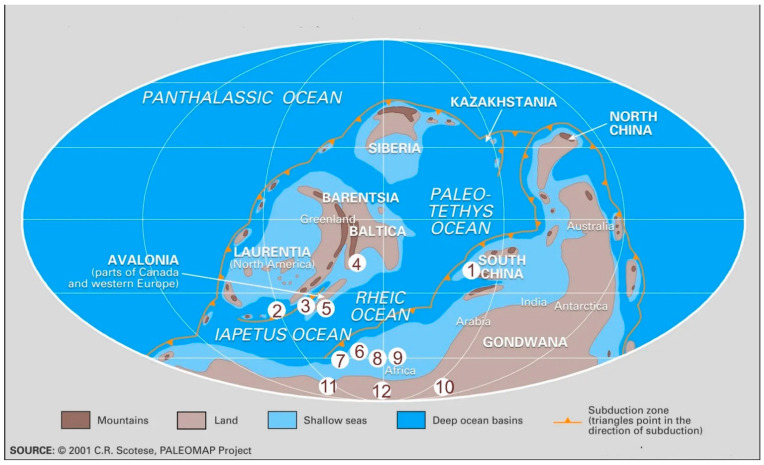
Reconstruction of palaeocontinents within Přídolí. Modified from (2001). 1. Yunnan, Sichuan. 2. USA. 3. Canada. 4. Estonia. 5. Ireland. 6. France. 7. Spain. 8. Algeria. 9. Libya. 10. Argentina. 11. Bolivia. 12. Brazil.

During the Přídolí, the climate was generally warmer than in stratigraphically older units. Siberia, Laurentia and Baltica formed a new “supercontinent”, Euramerica; e.g., Gondwana drifted over the South Pole and sea levels rose (Figure 1). Some areas near the equator were even characterised by evaporites, i.e., salt deposits [56]. The Přídolí epoch was a critical interval in the evolution of Earth’s biota. During this interval, powerful extinction events took place, with the consequent global reorganization of paleocommunities and expansion of new clades, which assumed dominance in the Devonian period. On land, bryophytes, early tracheophytes terrestrial fungi diversified, and in the marine realm, the first reefs appeared [don’t think this is correct- there were Cambrian reefs], and jawed and jawless fishes diversified.

The Silurian period, although short (only about 24 million years) was characterised by instability of the global carbon cycle and probable rapid changes in atmospheric pO2 and pCO2 [57,58,59,60,61,62]. At least five globally recognised carbon isotope excursions have been recognized, including the mid-Llandovery, early Wenlock, late Wenlock, late Ludlow and across the Silurian–Devonian boundary [63,64]. These rapid changes in the global carbon cycle have been associated with rapid changes in temperature [65,66]. If we characterize the occurrence of trilete spores and cryptospores in climatic belts it can help, based on our knowledge about in situ spores, with the occurrence of their parent plants within these belts, even though we did not record them there.

## 2. Material and Methods

Herein we focus on both plant megafossils and dispersed trilete spores and cryptospores. For plant megafossils, we provide an up-to-date list of all described Přídolí plant megafossil localities [2]. For dispersed trilete spores and cryptospores, we have used an extensive database of all records of Přídolí from [2] (Table 2). The database was assembled and analysed in 2011 and was updated with new unpublished data by one of the co-authors (P.S.), i.e., it brings in some new unpublished data. The data on stratigraphical and palaeogeographical occurrences of the Silurian miospore species have been treated by means of analysis in order to quantitatively analyse the similarities between assemblages belonging to defined palaeogeographical areas. The following palaeogeographical areas have been defined: ”southwestern Gondwana” (mainly North African localities); ”Peri-Gondwanan terranes” (corresponding to Western Europe, Bohemia, etc.); Avalonia (British Isles, part of northern France); Arabian Plate (including mainly Saudi Arabia and adjacent areas such as Iran and Iraq); Laurentia (corresponding to North America); ”Eastern Gondwana” (South America: Argentina, Paraguay, Bolivia); Baltica (Sweden, Norway, Batic states, northern Poland), South China Plate (localities belonging to the Yangtze Platform); and ”Northern Gondwana” (North Africa); additionally, South Africa is used as a separate palaeolocality, belonging to southern Gondwana.

### Přídolí Supergreenhouse-the Warmest Climate of the Silurian System

After the Late Ordovician (Hirnantian) glaciation, the Silurian began with a 7-million-year warming trend that lasted for most of the Llandovery (Llandovery Warm Trend) [66]. The start of the Wenlock (Sheinwoodian) is associated with a sharp drop in temperature and a biological crisis (Ireviken Bioevent) before the early Wenlock carbon isotope anomaly. After a slight warming in the middle Wenlock (Late Sheinwoodian to Early Homerian), there was a further drop in temperature in the upper Homerian. This cooling was also associated with a biological crisis (Lundgreni/Mulde bioevent) and a late Wenlock carbon isotope anomaly. The following period from the beginning of the Gorstian (Early Ludlow) to Přídolí is associated with a warming trend of about 7 million years. However, this long-term temperature trend was interrupted by a significant temperature decrease in the middle Ludfordian (late Ludlow) [65,66,67]. The marked decrease in sea surface temperatures (inferred from the positive shift in δ18Oapatite of at least 3%) recorded in the temperate areas of the Prague Basin of the Czech Republic and the Carnic Alps (peri-Gondwana), as well as in the tropical areas of Baltica (Laurussia) and Australia (Gondwana), coupled with a significant eustatic sea level decrease recorded in sequence stratigraphy on all corresponding palaeocontinents, points to glaciation (Mid-Ludfordian glaciation—see [27]—in polar and subpolar Gondwana. The Mid-Ludfordian glaciation is associated with the Ludfordian carbon isotope anomaly and was preceded by the Kozlowskii/Lau bioevent (see review in [27,28]). After this global cooling, the warming trend continued until Přídolí [65,66]. The end of Přídolí is associated with the onset of the Early Devonian cooling trend (sense [66]), which lasted about 30 million years until the Middle Devonian [66,68,69].

The Přídolí represents the warmest period of the entire Silurian, as confirmed by seawater temperatures derived from δ18O of brachiopod calcite shells and δ18O of conodont apatite [66]. However, the exact course of the temperature curve during the Přídolí is very poorly known. The main reason for this is the lack of subdivision of the Přídolí, which limits the possibility of stratigraphic correlations between individual palaeocontinents. In addition, in many areas the Přídolí strata are often not preserved or not studied in sufficient detail. As a result, data on seawater temperature in Přídolí are scarce [66].

The study of phosphatic microfossils such as conodonts and fish microremains (skin scales) from the Upper Silurian (Přídolí) of Lithuania revealed a short-term cooling event. In the mid-Přídolí within the *Ozarkodina eosteinhornensis* bizone or the *Ozarkodina remscheidensis* biozone, reflects a major cooling event that may have led to the formation of a high-latitude ice sheet and a glacio-eustatic sea-level fall [70]. A more detailed analysis has been carried out on conodont apatite from the Prague Basin of the Czech Republic [70,71]. New oxygen isotope data show a rapid climate warming and an increase in seawater temperatures of more than 8 °C in the mid-latitudes of northern Peri-Gondwana, followed by a strong cooling in the latest Přídolí and across the Silurian–Devonian boundary. The drastic climate change from a cold interval with strong cooling during the Early Přídolí followed by “supergreenhouse conditions” during the Late Přídolí transgredients graptolite zone probably caused dramatic extinctions and faunal turnover on a global scale [70,71,72].

Current knowledge of climate evolution during the late Přídolí shows that this period was the warmest in a long interval of about 60 million years from the Upper Ordovician to the Middle Devonian. Moreover, although the Přídolí only lasted about 4.5 million years, it was a period of rapid climatic change.

## 3. Palynology

### 3.1. Sheinwoodian Trilete Spores and Cryptospores

Records of Sheinwoodian trilete spores are known from tropical areas [16,73,74,75,76,77,78,79,80,81,82,83,84] and cool [85] temperate belts and consist of six species belonging to four genera (Table 3). The most abundant species are *Ambitisporites avitus* Hoffmeister and *A. dilutus* (Hoffmeister) Richardson [2].

Sheinwoodian cryptospore assemblages have only been described from the tropical belt [73,74,75,76,77,81,83,84] and yielded nine genera with 11 species. The most abundant species are *Laevolancis divellomedia* Chibrikova and *Tetrahedraletes medinensis* (Strother and Traverse) Wellman and Richardson [2]. The distribution of cryptospores and trilete spores is similar, as both groups reach their minimum numbers in the Sheinwoodian.

### 3.2. Homerian Trilete Spores and Cryptospores

During the Homerian, trilete spores from all climatic belts are recognized, and the diversity and abundance of spores increase. This corresponds to the first global key global events in the terestrialisation of early land plants after the Homerian glaciation [2,15]. The number of species and genera of trilete spores was about the same and relatively low until Homerian times (only about five species on average).

### 3.3. Gorstian Trilete Spores and Cryptospores

Cryptospore and trilete spore assemblages of the Gorstian have been described by [38,75,77,80,84,86,87,88,89]. The most common trilete spores are *Ambitisporites avitus* and *A. dilutus*; common cryptospores are *Archaeozonotriletes chulus* (Cramer) Richardson and Lister and *Cheilotetras caledonica* Wellman and Richardson. In general, there is a decrease in trilete spore taxa.

### 3.4. Ludfordian Trilete Spores and Cryptospores

Ludfordian cryptospore and trilete spore assemblages have been published by [38,78,79,80,84,86,87,88,89,90,91,92]. The most abundant trilete spores are of the genus *Ambitisporites* Hoffmeister, and the number of *Emphanisporites* McGregor increased. The most abundant cryptospores are *Archaeozonotriletes chulus*, *Laevolancis divellomediun* and the genus *Tetrahedraletes* (Strother and Traverse) Wellman and Richardson. The number of trilete spores increased slightly.

It is probable that some climatic change during the Gorstian caused a decrease in the trilete spore record, but the number of cryptospore species was the same as in the Homerian, i.e., this change affected only trilete spore producers and not Eophytidae, probably due to their different life strategies. The increase in the number of trilete spores from Ludlow to Přídolí is more pronounced (from 43 to 163) than that of cryptospores (from 33 to 51) within the Ludlow–Přídolí interval.

### 3.5. Přídolí Climatic Belts

The maximum number of both cryptospore and trilete spore species is in Přídolí, where cryptospores reached 43 species belonging to 21 genera and trilete spores and even 105 species belonging to 33 genera [2]. This second major global event has been documented, for example, by [2]. The previous Homerian event is only documented palynologically because macrofloral records are very rare and limited [2,40]; but the Přídolí event is well supported by the combination of both palynological and plant records.

The course of diversification of cryptospores is specific. Cryptospores reach their minimum as trilete spores in the Sheinwoodian and increased after the Homerian glaciation. The number of cryptospore species in the Gorstian is the same as in the Homerian, whereas trilete spores decreased significantly in the Gorstian. The general trend after the Gorstian, i.e., during Ludlow and Přídolí, is similar; cryptospores increased, but not as much as trilete spores. It gives evidence of/it implies different life strategies and ecological needs of eophytes and early land plants [2]. Ref. [54] showed different occurrences of cryptospores and trilete spores within the Sandbian–Gorstian interval. Cryptospores reached their maximum much earlier within the Hirnantian and Rhuddanian, but trilete spores have minimal numbers (only about five species on average) until the Sheinwoodian.

#### 3.5.1. Cryptospores in Tropical Climatic Belt

Cryptospores are represented by 10 genera with 13 species, and almost half (46%) of them (Table 2) are reported only from this climatic belt [2]. Cryptospore records are reported from Laurentia [74,93,94] and Avalonia [38,45,73,83,95,96,97,98,99,100]. The most common species are *Tetrahedraletes medinensis*, *Dyadospora murusattenuata* (Strother and Traverse) Wellman and Richardson, *Laevolancis divelomedium* and the genus *Artemopyra* Burgess and Richardson.

#### 3.5.2. Cryptospores in Temperate Climatic Belt

The number of cryptospore taxa is higher, with 13 genera and 21 species (Table 3). More than a third (38%) of these are restricted only to this climatic belt. The cryptospore records come from southern Peri-Gondwana [90,91,92,93,94,95,96,97,98,99,100,101,102,103,104,105,106,107,108,109], i.e., Armorica and Iberia. The most commonly recorded genera are *Dyadospora* (Strother and Traverse) Burgess and Richardson, *Pseudodyadospora* Johnson, *Quadrisporites* Hennelly and the genus *Hispanaediscus* (Cramer) Burgess and Richardson well diversified with four species.

#### 3.5.3. Cryptospores in Cool Climatic Belt

Cryptospores from the cool climate belt reach 12 genera with 25 species. More than half (60%) of these are restricted only to this belt. Cryptospores originated from southern Gondwana, including Libya [38,88,89,110,111,112], Argentina [113,114,115], Bolivia [116], Brazil and southern China [117,118]. The most common genera are *Artemopyra*, *Hispanaediscus* and especially *Cymbohilates* (Richardson) Breuer with five species.

The number of cryptospore taxa is highest in the temperate and cool climate belts, while the lowest number is in the tropical belt (only about half of those reported from only the cool climate belt). The number of endemic cryptospore taxa is highest in the cool belt, i.e., eophytids (cryptospore-producers) must have been more specialised for cooler climates than for warmer ones.

#### 3.5.4. Trilete Spores in Tropical Climatic Belt

Trilete spores described from the tropical belt are represented by 12 genera with 26 species (Table 3), a third (34%) of which are unique to this belt. Trilete spores are described from Laurentia [93,119], Avalonia [38,45,74,83,95,96,97,98,99,100] and Baltica [94].

#### 3.5.5. Trilete Spores in Temperate Climatic Belt

Trilete spores from the temperate belt comprise 24 genera and 70 species (Table 3). Nearly half (48%) of these occurred only in this belt. Records of trilete spores come from southern Peri-Gondwana, i.e., Armorica [96,101,102,103,104] and Iberia [87,90,91,92,105,106,107,108,109].

#### 3.5.6. Trilete Spores in Cool Climatic Belt

Records of trilete spores from the cool belt come from southern Gondwana, including Libya [38,88,89,111,112], Argentina [113,114,115], Bolivia [116], Brazil [112] and southern China [117,118]. This assemblage consists of 23 spore genera with 70 species, a third (34%) of which are unique to this region (Table 3).

The lowest number of trilete spore taxa is found in the tropics, and the temperate and cool belts have equal numbers. The number of endemic species, i.e., those found only in one climatic belt, varies from a third to almost half. Table 3 shows cryptospores, trilete spore and early land plant taxa recorded only in one climatic belt.

Both cryptospore producers (eophytids) and trilete spore producers may have been better adapted or more tolerant of cooler rather than warmer climates.

## 4. Palaeobotany

### Early Land Plants within Přídolí

The plant megafossil record from Přídolí is poor. We know 16 genera with 24 species [2], only from the tropical belt, and one questionable record of *Cooksonia* sp. from the temperate belt (Table 3). Based on our knowledge of in situ spores ([43,44,46]), we can roughly estimate the number of plant genera in the temperate and cool climate belts from the palynological record. A total of 33 spore taxa are recorded in the temperate climate belt, and it is possible that these spores are produced by 23 plant taxa. A total of 24 taxa of trilete spores from the cold belt could have been produced by 16 to 18 plant taxa. However, these numbers are highly hypothetical.

## 5. Discussion

Přídolí was the warmest Silurian period with temperatures much higher than in previous times. It is associated with a qualitative and quantitative increase in cryptospores and especially trilete spores, signaling a diversification and geographic spread of tracheophytes, a key event in the history of terrestrialisation.

The general pattern of distribution of cryptospore and trilete spore taxa within the Přídolí climatic belts is roughly comparable. The lowest diversity of both types of palynomorphs is in the tropical belt, where cryptospores and trilete spores reach their minimum number within Přídolí (13 and 26 species, respectively). There is a significant increase in the temperate belt, where trilete spores reach their maximum (72 species), comparable to the numbers in the cool climate belt. Cryptospores reach their maximum in the cool belt (25 species), while the number of trilete spores also remains significantly high (23 genera with 70 species). The pattern of distribution of taxa occurring only in one climatic belt, i.e., an increasing trend from the tropical to the cool climatic belt for cryptospores (7, 8 and 15 species). Species of trilete spores reported only in one climatic belt reach their maximum in the temperate belt (34) and decrease in the cool belt (24).

Dispersed cryptospores and trilete spores usually occur together and their different numbers in all climatic belts indicate that their producers, i.e., eophytes and early land plants, had different life strategies.

Another important feature is the different number of cryptospore and trilete spore species (Table 2) within the same climatic belt. The number of trilete spore taxa is more than three times higher than in the tropical belt. This prevalence of trilete spore diversity is, however, also clearly seen in the cool and temperate climatic zones (Table 3).

The number of trilete spore species (26) and early land plants fossils (24) is comparable in the tropical climate belt.

The situation with the occurrence of early land plants in Přídolí is constrained by the fact that almost all plant records come from the tropical belt (24 taxa); nothing is known from the cool belt and there is only one questionable record from the temperate belt (Table 2). This may indicate that conditions in the tropical belt were more favorable for plants fossilization than those in the temperate and cool belts. From the Devonian on, conditions were more favorable in cooler and especially wetter conditions for plant preservation (the Pennsylvanian equatorial lowlands were unusually cool). Especially given that the Pridoli was apparently quite warm, it seems unlikely that conditions in the tropics were more conducive to fossil preservation.

We know the affinities of some spores from studies of in situ spores, i.e., spores isolated directly from plant reproductive organs. Our knowledge of Upper Silurian plant producers is not perfect; that means compared, e.g., with that of Pennsylvanian spores [46]. Summaries of Silurian/Devonian in situ spores have been published by [43,44,46]. It is possible to propose a palynological grouping of Silurian and Early Devonian land plant spores and divide the Silurian/Early Devonian plants into three groups of rhyniophytes, two groups of zosterophytes, one group of trimerophytes and two groups of lycophytes, and propose the affinity of some plants to *Incertae sedis* on the basis of their in situ spores.

This means that we can compare the spores and plants recorded in the tropical climate belt in Přídolí, although the number of plant taxa (15 genera with 23 species) is higher than that of spores (6 genera with 9 species). Some spore taxa (*Scylaspora* Burgess and Richardson, *Cymbosporites* Allen and *Vermiverruspora* Beck and Strother) were produced by unknown parent plants. All others (*Ambitisporites* and *Synorisporites* Richardson and Lister) belong to rhyniophytes. Looking at the list of plants recorded from the tropical climatic belt, we can hypothetically estimate which spores were produced by them. We know in situ spores from only 10 plant taxa from the tropical belt. It is possible to estimate that these plants produced the spore genera *Ambitisporites*, *Apiculiretusispora* (Streel) Streel and *Retusotriletes* (Naumova) Streel.

It is possible to estimate that the parent plants of the spores recorded in the temperate zone might be produced by some rhyniophytes (*Apiculiretusispora*, *Retusotriletes*, *Streelispora* (Chaloner and Streel) Richardson and Lister, *Synorisporites*), trimerophytes (*Apiculiretusispora*, *Retusotriletes*), zosterophytes (*Retusotriletes*, *Calamospora* Schopf, Wilson and Bentall) and horneophytes (*Emphanisporites*). Out of 19 plant genera, in situ spores are known from only 7. Plant-produced spores from the temperate belt were some rhyniophytes, zosterophytes, trimerophytes and *Streelispora*. Of the 15 spore genera (with 24 species) described from the cool belt, we know producers of only 7, but it seems that rhyniophytes were predominant and some zosterophytes, trimerophytes and *Horneophyton* Barghoorn and Darrah were common.

In general, based on the dispersed spore record, we may estimate that plant assemblages of the tropical belt were dominated by rhyniophytes; trimerophytes probably outnumbered rhyniophytes in the temperate belt, and rhyniophytes again dominated within the cool belt. Surprisingly, however, the diversity of cryptospore producers was higher in the cool climatic belt, and trilete spore producers were more diverse in temperate and cool latitudes, compared to the tropical belt.

## 6. Conclusions

Generally, it is assumed that the tropics were evolutionary cradles throughout land plant evolutionary history [2,119]. Our analysis shows that the diversification of cryptospore and trilete-spore-producing plants took place in temperate and cool climatic belts, rather than the tropics. Diversity in the tropical belt is significantly lower compared to temperate and cool belts [2]. A similar pattern was reported for Late Silurian–Early Devonian palynomorphs [119]. Based on quantitative data from our database, it is evident that during the biggest Lower Paleozoic (Přídolí) event for cryptospore and trilete-spore-producing plants the diversification of land plants was much lower close to and at the equator compared to temperate and cool climatic belts. The diversity of the tropical belt is significantly lower compared to the situation in more diverse temperate and cool belts [2]. Similar results with higher occurrences of palynomorphs within cooler climatic belts from the Late Silurian–Early Devonian are confirmed in a simultaneously published paper [119].

Přídolí is characterised by a rapidly increasing number of cryptospores and especially trilete spores, perhaps because it was the warmest interval in the Silurian. Cryptopore producers (eophytes) that occur only in one climatic belt were most abundant in the cool belt, and autochtonous trilete-spore producers reached the highest numbers in the temperate belt. From the dispersed spore record, it is possible to estimate that the plant assemblages of the tropical belt were dominated by rhyniophytes, trimerophytes probably outnumbered rhyniophytes in the temperate belt, and rhyniophytes again dominated within the cool belt. Results show in which climatic areas the second global event originated and how it was important especially for the evolution of early vascular land plants. This might indicate an early land plants adaptation to cold continental conditions as envisaged by the cryogenian initiation of plant terrestrialization hypothesis [11].

## Figures and Tables

**Table 1:** Summary of main Silurian evolutionary morphological innovations.

Stage/Series	Palynological Event	Main Character
Přídolí	Diversification of crassitate, distally equatorially multisculptured miospores	Diversification and proliferation of murornate equatorially crassitate miospores, incoming of apiculate curvaturate forms and hilate dyads
	Murornate, apiculate patinate miospores	Diversification of distally reticulate patinate and foveolate sculptured miospores. Incoming of tripapillate and equatorially crassitate murornate forms and hilate dyads
Ludfordian	Patinate proximally hilate with distal faint muri miospores	Diversification of variably sculptured sculptured patinate, crassitate, cingulate and radially ribbed sporomorphs
Gorstian	Apiculate, patinate with faint radial muri miospores	Incoming of distally murornate miospores with radially ribbed patinate pattern. Consistent of hilate cryptospores and tetrads
Homerian	Granulate, apiculate and crassitate miospores	Incoming of proximal radial muri an dequatorial radial thickenings and distal grana and verrucae
	Murornate, verrucate, crassitate and patinate miospores	Proliferation of verrucate, murornate emphanoid forms and patinate miospores with distal radial muri including verrucate cryptospores
	Murornate and verrucate miospores	Dominance of distally apiculate and verrucate crassitate and cingulate miospores. Persistence of hilate cryptospores
	Hilate monads	Incoming of patinate miospores included dyads and tetrads
Sheinwoodian	Patinate, proximally hilate laevigate miospores	Incoming of hilate miospores, equatorially crassitate miospores, tetrads and dyads. First monolete miospores
Telychian	Crassitate, distally laevigate miospores	Incoming of laevigate crassitate miospores and some permanent tetradas and dyads and persistence of cryptospores

**Table 2:** The number of cryptospores and trilete spore taxa including autochthonous ones in tropical, temperate and cool climatic belts.

Climatic Belt	Cryptospore Taxa Generally	Autochthonous Cryptospore Taxa	Trilete Spore Taxa Generally	Autochthonous Trilete Spore Taxa	Plant Taxa Generally (= Autochthonous)
Tropical	13	7	26	9	24
Temperate	21	8	72	34	0
Cool	25	15	70	24	0

**Table 3:** Autochtonous (i.e., occur only in one climatic belt) trilete spore and early land plant taxa.

Climatic Belt	Trilete Spores	Early Land Plants
Tropical	*Ambitisporites capitaneus*, *A. parvus*, *A. warringtonii*, *Cymbosporites echinatus*, *Retusotriletes charulatus*, *R. simplex*, *Scylaspora asperverruca*, *Synorisporites labeonis*, *Vermiverruspora cotter*	*Cooksonia pertoni*, *C. cambrensis*, *C. hemisphaerica*, *Cooksonia* cf. *hemisphaerica*, *C. bohemica*, *Cooksonia* sp., *Hollandophyton colliculum*, *Caia langii*, *Pertonella dactylethra*, *Salopella xinjiangensis*, *S.* sp., *Psilophytites* sp., *Tortilicaulis transwalliensis*, *Steganotheca striata*, *Eorhynia*, *Lycopodolica*, *Cooksonella* sp., ?*Baragwanathia* sp., *Taeniocrada* sp., *Jugumella burubaensis, Zosterophyllum qujingense, Zosterophyllum* sp., *Junggaria spinosa*
Temperate	*Anapiculatisporites isidori*, *A. terciensis*, *A. ventae*, *Apiculiretusispora arcidecus*, *A. microconus*, *A. plicata*, *A. toriensis*, *Archaicusporites asturicus*, *A. torrestionensis*, *Calamospora microrugosa*, *Concentricosisporites borbullatus*, *Convolutispora quitidae*, *Coronaspora infraornata*, *C. mariae*, *C. primordiale*, *C. subornata*, *Cymbosporites catillus*, *Emphanisporites disformis*, *E. perfilum*, *Chelinospora canistata*, *Ch. lavidensis*, *Ch. media*, *Knoxisporites riondae*, *Leiotriletes pyramidalis*, *L. socorridus*, *L. titanicus*, *Punctatisporites punctatus*, *Retialetes legionis*, *Retusotriletes aureolatus*, *R. coronatus*, *Scylaspora elegans*, *Stenozonotriletes pumillus*, *Streelispora granulata*, *Synorisporites lobatus*	
Cool	*Aneurospora geikiei*, *Synorisporites richardsonii*, *Apiculiretusispora perfecta*, *Brochotriletes foveolatus*, *Cymbosporites sparseus*, *Dictyotriletes gorgoneus*, *Emphanisporites multicostatus*, *Chelinospora retorrida*, *Iberospora noninspisatosa*, *Perotrilites laevigatus*, *Retusotriletes amazonensis*, *R. delicatus*, *R. maccullocki*, *R. maculatus*, *R. pychovii*, *Scylaspora distincta*, *S. downiei*, *S. chartulatus*, *S. kozlica*, *S. radiata*, *Segestrespora membranifera*, *Synorisporites libycus*, *S. papillensis*, *Verrucosisporites devonicus*	

## Data Availability

Palynological data are available in database stored in the University of Liege, Belgium.
